# Clinical Characteristics and Gene Mutations of Hereditary Spherocytosis in 59 Chinese Children

**DOI:** 10.1002/mgg3.70188

**Published:** 2026-02-21

**Authors:** Yuzhuopu Li, Yang Wang, Tao Liu, Li Xiao, Lan Huang, Yongjie Zhang, Yan Xiang, Jie Yu

**Affiliations:** ^1^ Department of Hematology and Oncology Children's Hospital of Chongqing Medical University, National Clinical Research Center for Child Health and Disorders, Ministry of Education Key Laboratory of Child Development and Disorders, Chongqing Key Laboratory of Pediatrics, Children's Hospital of Chongqing Medical University Chongqing China; ^2^ Hematology Department of Binzhou Medical College Affiliated Hospital Binzhou China; ^3^ Hebei Key Laboratory of Panvascular Diseases Chengde China

**Keywords:** *ANK1*, gene mutations, genetic analysis, hereditary spherocytosis, mutation spectrum, *SPTB*

## Abstract

**Background and Aim:**

Hereditary spherocytosis (HS) is a common disease in hereditary hemolytic anemia. Advancements in sequencing technology have enabled the identification of a growing number of mutation sites associated with HS. This study analyzed the clinical characteristics and gene mutations of HS in our center.

**Methods:**

Retrospective collection of data on 59 Chinese pediatric patients with HS admitted to the Hematology Department of Chongqing Medical University Affiliated Children's Hospital from 2013 to 2022. Second‐generation gene sequencing was performed on participants, with verification of detected variants using Sanger sequencing. Data analysis was conducted using various databases, and statistical methods were used for differential analysis.

**Result:**

We collected clinical data of 59 Chinese children with HS phenotype, including 27 males (45.8%) and 32 females (54.2%), all unrelated. The age of onset ranged from 0 to 180 months, with a median age of 60 months. Our study found that *ANK1* and *SPTB* gene mutations were the primary causes of HS, with missense and frameshift mutations being the most common. De novo mutations were present in 37 (62.7%) patients, while the remaining mutations were inherited. We noted a higher proportion of females (*p* = 0.032) and lower total bilirubin levels (*p* = 0.014) in patients with multiple gene mutations. Patients with *ANK1* gene mutations experienced more severe anemia compared to those with *SPTB* gene mutations (*p* = 0.041). Additionally, there were significant differences in mean corpuscular hemoglobin concentration (MCHC) between different mutation types (*p* = 0.036), indicating lower MCHC levels in the missense mutation group. No differences in clinical phenotypes were observed among different structural domains of *ANK1* and *SPTB* mutations. Splenectomy significantly alleviated the symptoms in HS patients.

**Conclusion:**

We identified unique genetic and clinical characteristics mutations of HS in Chongqing, China. These findings expand the mutation spectrum of HS and have implications for early diagnosis and treatment of the disease.

## Graphical abstract

Summary of the causative genes' spectrum of 59 Chinese HS Children patients. The schematic diagram of ankyrin, β‐spectrin, α‐spectrin, and band three protein domains with ANK1, SPTB, SPTA1, and SLC4A1 mutations. Distribution of mutations in the exons of ANK1 and SPTB genes.

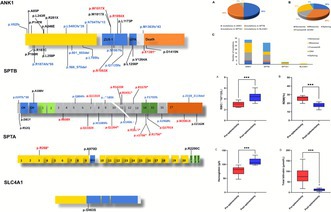

## 1 Introduction

Hereditary spherocytosis (HS) is a genetic, nonimmune hemolytic disorder characterized by jaundice, hemolysis, splenomegaly, and gallstones as the main clinical manifestations (Butorac et al. 2018; Zamora and Schaefer 2023). The clinical presentations of HS patients vary, with mild HS children often having no significant clinical symptoms, while severe HS children require frequent blood transfusions to sustain life. Some HS patients can experience hemolytic crises, which can be life‐threatening (Yamamoto et al. 2022). The pathogenesis of HS is based on mutations in the *ANK1*, *SPTB, SLC4A1, EPB42*, and *SPTA1* genes, which encode red blood cell membrane skeleton proteins, leading to the spherical deformation of red blood cells and ultimately causing hemolysis (Park et al. 2016) (Li et al. 2022). The severity of HS is directly related to the loss of membrane surface area (Iolascon et al. 2019). Studies have shown that the prevalence of HS is approximately 1/2000 in Northern Europe (Bolton‐Maggs et al. 2012). In China, the incidence of HS is approximately 1/100,000 (Wang et al. 2015). The low number of HS cases and high genetic and clinical heterogeneity pose challenges for diagnosis and treatment. With the development and popularization of high‐throughput genomic sequencing, the emergence of large‐scale genetic data has followed. High‐throughput and accurate gene sequencing technologies have now become powerful tools for assisting HS diagnosis (Wang et al. 2021; Yang et al. 2019). Therefore, the establishment of an auxiliary diagnostic system and a gene‐clinical database based on the relationship between genotypes and clinical phenotypes is of great significance. However, it is regrettable that the full spectrum of genetic mutations in HS has not yet been fully described.

In this study, we conducted an analysis of clinical data from 59 Chinese patients with HS and explored in depth their clinical and genetic mutation characteristics. Our findings contribute to the delineation of the genetic mutation spectrum in real‐world HS patients and provide data support for genetic counseling of HS patients.

## 2 Methods

### 2.1 Patient Information

This study is retrospective research. Clinical data of Chinese pediatric patients with HS (Hereditary Spherocytosis) admitted to the outpatient and inpatient departments of the Hematology Department of Chongqing Medical University Affiliated Children's Hospital from 2013 to 2022 were collected. The diagnostic criteria for HS are as follows: (1) Having a family history, exhibiting hemolysis and splenomegaly, with high mean corpuscular hemoglobin concentration (MCHC) and reticulocyte (RET) ratio, a significant amount of spherocytes in the peripheral blood, and a positive OFT (osmotic fragility test) or EMA (Eosin‐5′‐maleimide) binding test can confirm a clinical diagnosis; (2) Without a family history, and atypical laboratory results (such as negative OFT and not many spherocytes in peripheral blood, but positive EMA binding test) can be considered as a clinical suspicion, with a positive pathogenic gene mutation confirming the diagnosis; (3) No family history, laboratory results indicating HS, but no pathogenic gene mutations found or the pathogenicity of variant genes is unknown in genetic analysis; such patients can still be diagnosed based on the efficacy of splenectomy and the exclusion of other hemolytic diseases. All patients were excluded from having glucose‐6‐phosphate dehydrogenase (G‐6‐PD) deficiency, thalassemia, hemoglobinopathies, and immune hemolytic anemia.

Collected clinical data include: (1) patient's gender, age, family history; (2) complete blood count (CBC), reticulocyte count (RET), total bilirubin (TBIL), transfusion history, presence of splenomegaly, splenectomy status, and presence of gallstone. CBC samples were collected before transfusion and before splenectomy, avoiding periods of various infections; TBIL measurement for patients with concurrent cholelithiasis was conducted avoiding periods of biliary obstruction.

In the subsequent statistics, we will unify the compound heterozygosity and the co‐inheritance of multiple variables into the compound mutations group for ease of statistics.

### 2.2 Genetic Testing and Analysis

Peripheral blood genomic DNA was extracted using DNA extraction kits from Beijing Tiangen Biochemical Technology Co. Ltd., and the QIAamp DNA Mini Kit from Qiagen, Germany. The Next‐Generation Sequencing (NGS) panel targeted coding exons and key intronic regions of genes related to RBC membrane disorders, enzyme deficiencies, congenital dyserythropoietic anemia, hemoglobinopathies, and bilirubin metabolism. DNA libraries were constructed using the GenCap Custom Kit (MyGenostics, Beijing), which focused on genes such as *ANK1*, *SPTB*, *SLC4A1*, and *SPTA1* (CNVs were inferred from NGS depth‐of‐coverage ratios using CODEX2^v1.40.0^, with validation by MLPA for putative deletions/duplications). Biotin‐labeled capture probes (80–120‐mers) were employed to encompass all exons within nonrepeat regions. The average gene coverage for the targeted areas was 94.86%, with an average sequencing depth of 436.75×. Additionally, 90.14% of the targeted regions had a coverage of more than 30×, and 60.29% had over 200× coverage. Genetic variants were analyzed using the Human Gene Mutation Database (https://www.hgmd.cf.ac.uk/ac/index.php), ClinVar (http://www.ncbi.nlm.nih.gov/clinvar), and the dbSNP database (https://www.ncbi.nlm.nih.gov/snp). The pathogenicity of mutations was predicted using the Protein Variation Effect Analyser (PROVEAN, http://provean.jcvi.org) and MutationTaster (https://www.mutationtaster.org), and the identified variant sites were comprehensively analyzed according to the guidelines and classification criteria of the American College of Medical Genetics (ACMC, Richards et al. 2015) to determine their pathogenicity. Finally, variant calling was performed using GATK HaplotypeCaller (v4.2) with default parameters, followed by joint genotyping and VQSR filtering.

### 2.3 Statistical Methods

Statistical analysis was performed using SPSS 27. All tests were two‐tailed, and a *p*‐value < 0.05 was considered statistically significant. Data were described using median (range) and percentages. The chi‐square test was used for analyzing categorical variables. For normally distributed variables, *t*‐test or one‐way analysis of variance (ANOVA) was used for between‐group comparisons. For nonnormally distributed variables, the Mann–Whitney *U* test or Kruskal–Wallis *H* test was used for between‐group comparisons.

## 3 Results

### 3.1 Clinical Characteristics of Patients With HS

We collected clinical data from 59 Chinese children with HS and summarized them in Table 1. The patients exhibited varying degrees of anemia, with eight cases classified as mild anemia (90 g/L < Hb ≤ 120 g/L), 45 cases as moderate anemia (60 g/L < Hb ≤ 90 g/L), and six cases as severe anemia (Hb ≤ 60 g/L). Among them, there were 27 male patients (45.8%) and 32 female patients (54.2%), all of whom were unrelated. The age at onset ranged from 0 to 180 months, with a median age of 60 months. Among the patients, 14 cases (23.7%) had an onset age of less than 1 year. We found that among the 59 patients, 22 cases (37.3%) had a family history of inheritance, with 12 cases inheriting the mutation from their fathers and 10 cases inheriting it from their mothers. The remaining 37 cases (62.7%) had de novo mutations. The median levels of hemoglobin, mean corpuscular volume, mean corpuscular hemoglobin concentration, reticulocyte percentage, and total bilirubin were 78 g/L, 83.2 fL, 329 g/L, 8.2%, and 61.1 μmol/L, respectively, for all children. In addition, 48 patients (81.4%) received blood transfusion, 48 patients (81.4%) had splenomegaly, 12 patients (20.3%) underwent splenectomy, and 12 patients (20.3%) developed gallstones. Except for eight patients (P10, P18, P19, P24, P29, P30, P34, P44), peripheral blood smears of all other patients showed spherocytes. For more detailed clinical information, refer to Table S1.

**TABLE 1:** Clinical manifestations of 59 children with diagnosed HS mutations in China.

Characteristic	Mild (*n* = 8)	Moderate (*n* = 45)	Severe (*n* = 6)
Gender			
Male, *n*	6	19	6
Female, *n*	2	26	0
Month‐old at diagnosis; Med (range)	42 (3.2–84)	60 (0.33–180)	39 (0–135)
Family history, *n*	2	18	2
Hb (g/L), Med (range)	99 (93–105)	77 (61–90)	50 (30–60)
MCV (FL), Med (range)	81.6 (77.9–86.2)	83.9 (72.9–94.6)	79.4 (69.8–86.2)
MCHC (g/L), Med (range)	336 (324–348)	328 (281–363)	305 (234–333)
Retics (%), Med (range)	5.4 (1.4–8)	8.7 (1.2–26.1)	8.9 (4–52)
TBIL (μmol/L), Med (range)	39.6 (4–61.1)	66.3 (8.7–171.5)	60.4 (16.4–338.9)
Transfusion, *n*	6	36	6
Splenomegaly, *n*	5	39	4
Splenectomy, *n*	3	9	0
Gall stones	4	8	0
Month‐old of splenectomy; Med (range)	104 (102–161)	102 (62–180)	—

Abbreviations: Hb, hemoglobin; MCHC, mean corpuscular hemoglobin concentration; MCV, mean corpuscular volume; Med, median; TBIL, total bilirubin.

### 3.2 Mutational Spectrum of HS at Our Center

Based on the detected gene mutations in the 59 HS patients, a mutation spectrum specific to our center was plotted. There are a total of 53 patients with single‐gene mutations, including 25 patients with *ANK1* gene mutations, 25 patients with *SPTB* gene mutations, two patients with *SPTA1* gene mutations, and one patient with *SLC4A1* gene mutation. Additionally, there are six patients with compound mutations. Among the compound mutations, P10, P13, P23, and P25 carried two *ANK1* mutations each, P9 carried a *SPTA1* mutation combined with an *SPTB* mutation, and P36 carried three *SPTB* mutations (During genetic testing, we did not find the hypomorphic *SPTA1* alleles, namely alpha LELY and LEPRA.).

**FIGURE 1:**
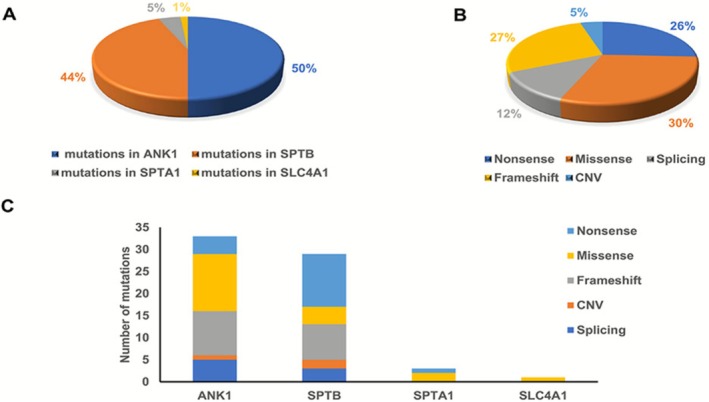
Summary of the causative genes' spectrum of 59 Chinese HS Children patients. (A) The distribution of four causative genes (blue‐*ANK1*, orange‐*SPTB*, grey*‐SPTA1*, yellow‐*SLC4A1*) mutations and their respective proportions. (B) Types of all mutations (dark blue‐Nonsense, orange‐Missense, grey‐Splicing, yellow‐Frameshift, light blue‐CNV) and their respective proportions. (C) Types of *ANK1, SPTB, SPTA1*, and *SLC4A1* mutations and the proportion of different mutation types (light blue‐Nonsense, yellow‐Missense, dark blue‐Splicing, grey‐Frameshift, orange‐CNV) in different mutated genes.

**FIGURE 2:**
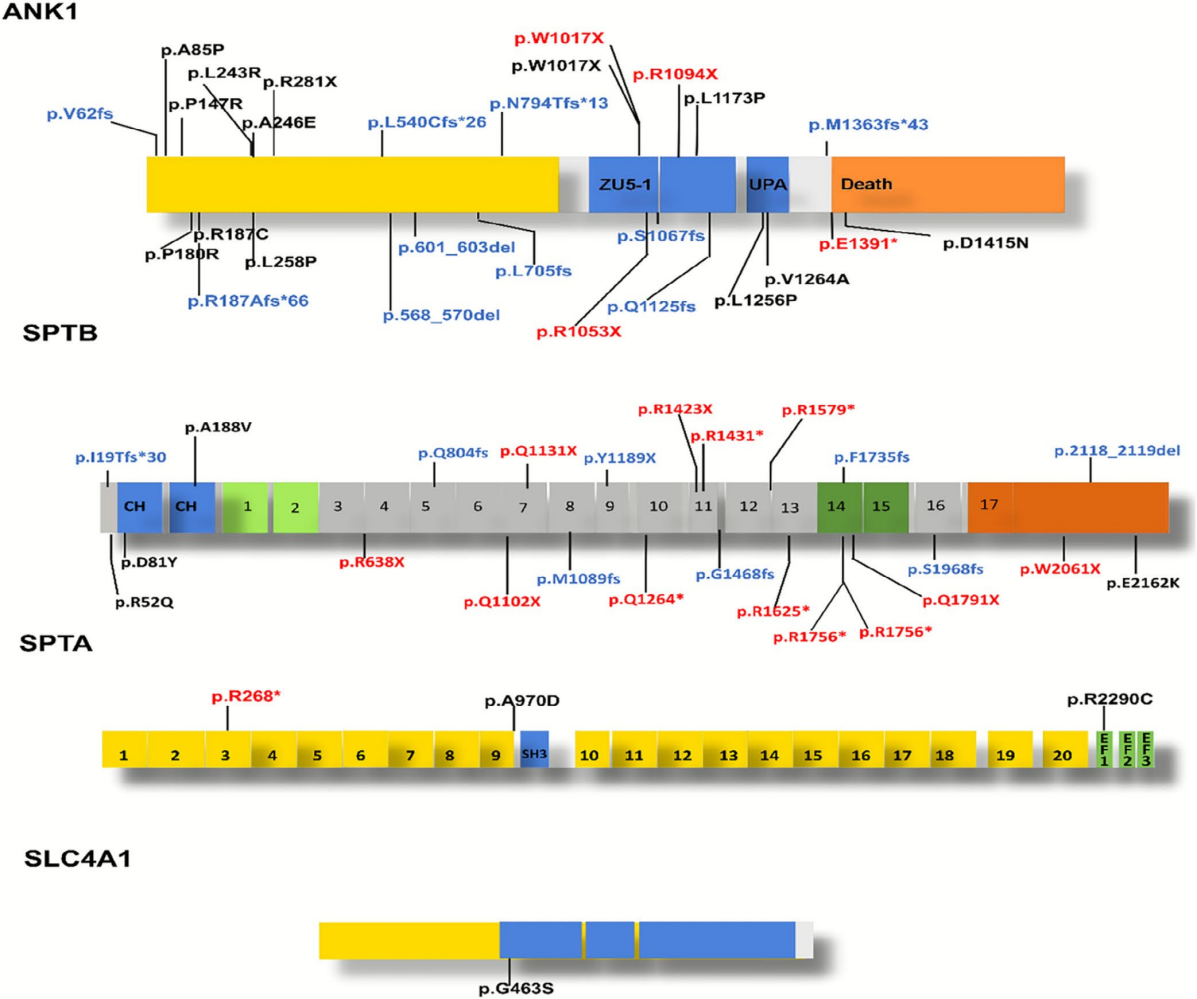
The schematic diagram of ankyrin, β‐spectrin, α‐spectrin, and band three protein domains with *ANK1, SPTB, SPTA1*, and *SLC4A1* mutations. (A) *ANK1* anchoring domain: The diagram highlights the different regions of *ANK1*. The yellow region represents the N‐terminal membrane protein binding domain, the blue region represents the central domain, and the orange region represents the C‐terminal regulatory region. (B) *SPTB* anchoring domain: The diagram illustrates the various regions of *SPTB*. The blue region represents the actin binding domain, the light green region represents the dimerization domain, the gray region represents parts of the spectrin repeats domain, the dark green region represents the ankyrin binding domain, and the orange region represents the tetramerization domain. (C) *SPTA1* anchoring domain: The diagram depicts the different regions of *SPTA1*. The yellow region represents the spectrin repeats domain, the blue regions represent the src homology 3 domains, EF1 and EF2 indicate the EF‐hand domain, and EF3 represents the Ca^2+^ insensitive EF hand domain. (D) *SLC4A1* anchoring domain: The diagram shows the N‐terminal cytoplasmic domain (yellow region) binding with *ANK1* and the C‐terminal domain (blue region) spanning the lipid bilayer involved in anion transport. The different mutation types are labeled with different colors: Black for missense mutations, red for nonsense mutations, and blue for frameshift mutations.

**FIGURE 3:**
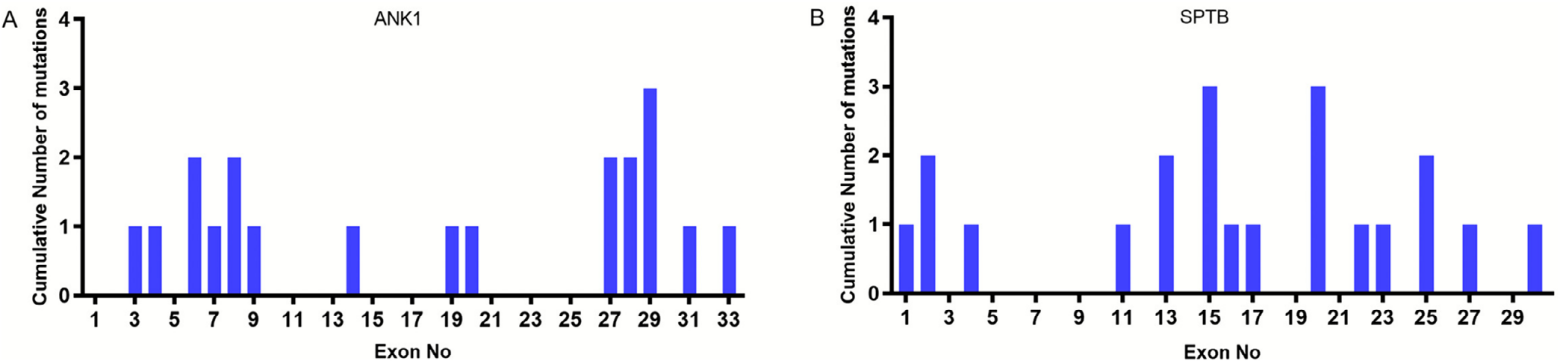
Distribution of mutations in the exon of *ANK1* and *SPTB* gene. (A) Cumulative number of mutations in each exon of the *ANK1* gene. (B) Cumulative number of mutations in each exon of the *SPTB* gene.

A total of 66 pathogenic mutation sites were detected in this study. The number of mutations in *ANK1, SLC4A1, SPTA1*, and *SPTB* genes was 33 (50%), 29 (44.5%), 3 (4.5%), and 1 (2%), respectively (Figure 1A). *SPTB* and *ANK1* were the main genes mutated in Chinese HS patients. Among these mutations, there were 20 missense mutations (30.3%), 18 frameshift mutations (27.2%), 17 nonsense mutations (25.8%), 8 splice site mutations (12.1%), and 3 copy number variations (CNVs) (5%) (Figure 1B). Missense mutations were the most common type of mutation in the *ANK1* gene (39.3%), whereas nonsense mutations were the most common type in *SPTB* gene mutations (41.3%) (Figure 1C). In this study, a total of 66 variants were detected, and among them, 55 variants that impact structural domains, excluding 8 splice site mutations and 3 CNV mutations, are displayed in Figure 2. The specific locations of mutations in the exons of the *ANK1* and *SPTB* genes were summarized in Figure 3. Mutations in the *ANK1* gene were mainly found in exons 27, 28, and 29 (Figure 3A), while most of the *SPTB* gene mutations were located in exons 15, 20, and 25 (Figure 3B).

### 3.3 The Correlation of Gene With Clinical Traits

#### 3.3.1 Comparison of Clinical Characteristics Between Patients With Single Mutation and Patients With Multiple Mutations

The clinical characteristics of patients with single mutations and patients with multiple mutations are summarized in Table 2. There was a significant difference in gender distribution between the two groups, with a higher proportion of females in the compound mutation group (*p* = 0.032). There was also a significant difference in total bilirubin levels between the single mutation group and the multiple mutations group, with higher levels observed in the single mutation group (*p* = 0.014). There were no significant differences between the two groups in terms of age, family history, red blood cell‐related parameters, transfusion rate, splenomegaly, and splenectomy (*p* > 0.05).

**TABLE 2:** Comparison of clinical characteristics between single‐gene mutation and compound mutation groups.

Gene	Single‐gene mutation	Compound mutations	*p*
Gender, *n*			
Male, *n*	27	0	0.032
Female, n	26	6	
Month‐old at diagnosis; Med (range)	60 (0.3–180)	29 (0–101)	0.214
Family history, *n* (%)	36	3	0.379
Hb (g/L), Med (range)	79 (30–103)	72 (60–88)	0.668
MCV (FL), Med (range)	83.2 (22.9–93.6)	84.7 (72.9–90.4)	0.89
MCHC (g/L), Med (range)	331 (234–363)	328 (305–336)	0.374
Retics (%), Med (range)	8.2 (1.2–52)	8.7% (1.5–21.6)	0.965
TBIL (μmol/L), Med (range)	62.7 (16.9–171.5)	41.0 (25.4–338.9)	0.014
Transfusion, *n* (%)	45	4	0.822
Splenomegaly, *n* (%)	44	5	1
Splenectomy, *n* (%)	11	1	0.121

Abbreviations: Hb, hemoglobin; MCHC, mean corpuscular hemoglobin concentration; MCV, mean corpuscular volume; Med, median; TBIL, total bilirubin.

#### 3.3.2 The Relationships Between the Single Genes and Clinical Factors Were Analyzed

##### 3.3.2.1 Comparison of Clinical Characteristics Between Different Mutated Genes

To investigate the correlation between clinical phenotypes and genotypes in HS patients, we integrated the clinical symptoms of patients carrying *ANK1* and *SPTB* gene mutations, as shown in Table 3. The results showed that there were no significant differences between the two groups of patients in terms of gender, age, family history, hemoglobin levels, MCV, MCHC, reticulocyte levels, total bilirubin levels, transfusion rate, splenomegaly, and splenectomy (*p* > 0.05) (It should be noted that due to the small sample size of patients with *SLC4A1* and *SPTA1* gene mutations, they were not included in the statistical analysis of this section.).

**TABLE 3:** Comparison of clinical symptoms in children with different membrane protein gene mutations.

Gene	*ANK1* (*n* = 25)	*SPTB* (*n* = 25)	*p*
Gender			
Male, *n*	12	12	1
Female, *n*	13	13	
Month‐old at diagnosis; Med (range)	55 (1.9–146)	53 (0.33–180)	0.383
Family history, *n*	9 (34.5%)	6 (33.3%)	0.355
Hb (g/L), Med (range)	75 (30–103)	79 (61–102)	0.076
MCV (FL), Med (range)	82.3 (69.8–93.6)	84 (72.9–90.4)	0.321
MCHC (g/L), Med (range)	318 (234–342)	338 (312–363)	0.347
Retics (%), Med (range)	9.0 (1.3–52)	7.7% (1.2–19.1)	0.078
TBIL (μmol/L), Med (range)	61.1 (16.4–164.2)	65.8 (16.4–171.5)	0.571
Transfusion, *n*	21	21	1.000
Splenomegaly, *n*	22	20	0.440
Splenectomy, *n*	7	3	0.157
Gall stones	7	5	0.508

Abbreviations: Hb, hemoglobin; MCHC, mean corpuscular hemoglobin concentration; MCV, mean corpuscular volume; Med, median; TBIL, total bilirubin.

##### 3.3.2.2 To Analyze the Clinical Phenotypic Differences of *ANK1* and *SPTB* Gene Mutations in Their Different Structural Domains

We performed a comparative analysis of the differences in clinical phenotypes between *ANK1* gene mutations and *SPTB* gene mutations located in different structural domains. Tables S2 and S3, respectively show the median values (range) of different clinical parameters, such as hemoglobin, MCV, MCHC, reticulocytes, and total bilirubin, in patients with *ANK1* gene mutations and *SPTB* gene mutations located in different structural domains. It can be observed that there were no statistically significant intergroup differences in red blood cell‐related parameters and bilirubin levels due to different mutation domain locations (It should be noted that the C‐terminal regulatory region of *ANK1* and the dimerization domain of *SPTB* were not included in the statistical analysis of this section due to insufficient data.).

##### 3.3.2.3 Analysis of Clinical Characteristics and Laboratory Parameters of HS Patients With Different Types of Gene Mutations

In this study, we compared the differences between different mutation types, such as nonsense mutations, missense mutations, splice site mutations, frameshift mutations, and CNV, in relation to clinical characteristics and laboratory parameters. Table 4 for detailed information. The results showed that there was a significant difference in MCHC levels among different mutation types (*p* = 0.036), with the Missense group having the lowest MCHC levels. However, there were no statistically significant differences in the other parameters.

**TABLE 4:** Comparison of different gene mutation types and routine laboratory.

Types of gene mutations	Nonsense	Missense	Splicing	Frameshift	CNV	*p*
Gender						
Male, *n*	6	10	5	5	1	0.158
Female, *n*	8	5	1	10	2	
Month‐old at diagnosis; Med (range)	35.5 (0.53–135)	84 (2.9–180)	49 (1.9–131)	85 (0.33–152)	47 (46–63)	0.671
Family history, *n*	3	8	0	4	2	0.074
Hb (g/L), Med (range)	74 (30–100)	75 (47–105)	85 (51–102)	77 (61–98)	86 (83–88)	0.615
MCV (FL), Med (range)	82 (69.8–88.9)	83.9 (75.9–94.6)	82.6 (77.5–93.6)	83.7 (72.9–88.8)	83.2 (78.6–84.9)	0.377
MCHC (g/L), Med (range)	335 (312–363)	324 (234–340)	323 (302–348)	335 (311–362)	321 (320–325)	0.036
Retics (%), Med (range)	8.3 (2.2–52)	10.7 (1.4–26.1)	7.2 (1.2–19.4)	7.7 (1.3–15.1)	8.5 (6.2–9)	0.911
TBIL (μmol/L), Med (range)	64.6 (16.4–171.5)	53.1 (4–157.6)	58 (48–164.2)	73 (22.8–103.9)	23.3 (18.2–79)	0.374
Transfusion, *n*	12	12	5	13	2	0.958
Splenomegaly, *n*	12	12	4	13	2	0.784
Splenectomy, *n*	0	5	1	5	1	0.173

Abbreviations: Hb, hemoglobin; MCHC, mean corpuscular hemoglobin concentration; MCV, mean corpuscular volume; Med, median; TBIL, total bilirubin.

##### 3.3.2.4 Association Between Mutation Genes, Mutation Types, and Inheritance Patterns With Severity of HS Anemia

In this study, a comparison of mutated genes and mutation patterns of different membrane proteins with clinical symptoms in children was conducted. The distribution of characteristics among children with mild, moderate, and severe clinical symptoms was analyzed.

For mutated genes, *ANK1* was found in two cases of mild symptoms, 18 cases of moderate symptoms, and five cases of severe symptoms. *SPTB* was present in five cases of mild symptoms, 20 cases of moderate symptoms, and was not observed in cases of severe symptoms. The *p*‐value for the gene characteristic was calculated to be 0.041, indicating a significant difference among the severity groups.

Regarding mutation types, the distribution of nonsense, missense, splicing, frameshift, and CNV mutations was assessed. Nonsense mutations were identified in two cases of mild symptoms, 10 cases of moderate symptoms, and two cases of severe symptoms. Missense mutations were observed in two cases of mild symptoms, 11 cases of moderate symptoms, and two cases of severe symptoms. Splicing mutations were found in one case of mild symptoms, four cases of moderate symptoms, and one case of severe symptoms. Frameshift mutations were present in three cases of mild symptoms, 12 cases of moderate symptoms, and were absent in cases of severe symptoms. CNV mutations were not detected in cases of mild symptoms, were found in three cases of moderate symptoms, and were absent in cases of severe symptoms. The *p*‐value for the mutation type characteristic was calculated to be 0.880, indicating no significant difference among the severity groups.

In terms of inheritance patterns, de novo mutations were identified in six cases of mild symptoms, 27 cases of moderate symptoms, and four cases of severe symptoms. Paternal inheritance was observed in two cases of mild symptoms, 10 cases of moderate symptoms, and was not present in cases of severe symptoms. Maternal inheritance was not found in cases of mild symptoms, was observed in eight cases of moderate symptoms, and in two cases of severe symptoms. The *p*‐value for the inheritance characteristic was calculated to be 0.410, indicating no significant difference among the severity groups (Tables S4 and S5).

#### 3.3.3 Treatment Effect Before and After Splenectomy

**FIGURE 4:**
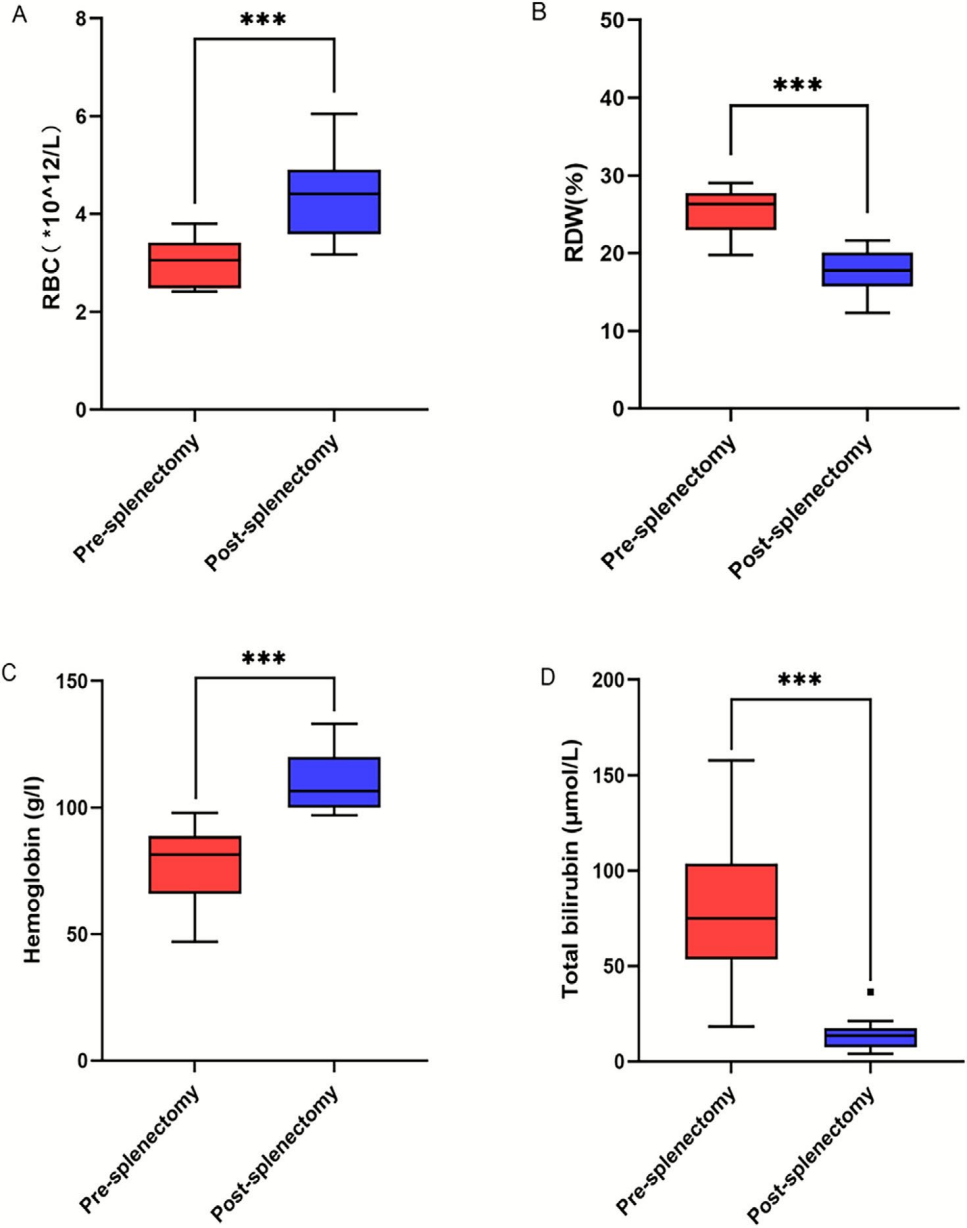
Change in (A) RBC, (B) RDW, (C) Hemoglobin, (D) Total bilirubin in response to splenectomy. Shown are medians and IQR (Interquartile Range). We observed some outliers, represented by squares, in the box plot (in D), indicating significant differences in vehicle outcomes for individual patients compared to the majority.

Among the 59 HS patients, 12 patients underwent splenectomy at an average age of 96 months. The changes in blood parameters before and after splenectomy are summarized in Table 5. We found that after splenectomy, there were significant improvements in red blood cell count, hemoglobin levels, red cell distribution width, and total bilirubin levels (*p* < 0.001), indicating that splenectomy can significantly improve the symptoms of HS patients (Figure 4).

**TABLE 5:** Changes in blood parameters before and after splenectomy.

Patient ID	TBIL (μmol/L)			Hemoglobin (g/L)			RDW			RBC		
	Pre‐op	Post‐op	*D*	Pre‐op	Post‐op	*D*	Pre‐op	Post‐op	*D*	Pre‐op	Post‐op	*D*
12	149	36.4	112.6	89	121	−32	27.6	20	7.6	3.56	4.82	−1.26
14	18.2	16	2.2	88	105	−17	23.2	17.4	5.8	3.45	4.45	−1
19	72.8	16.8	56	47	101	−54	41	15.3	25.7	2.43	3.46	−1.03
21	61.1	13.2	47.9	98	116	−18	23.4	16.7	6.7	3.8	4.07	−0.27
25	25.4	7.8	17.6	88	100	−12	19.8	12.3	7.5	3.32	4.76	−1.44
26	157.6	13.9	143.7	89	108	−19	25.8	19.3	6.5	3.19	4.99	−1.8
27	80.8	21.23	59.57	61	97	−36	27.8	20.1	7.7	2.44	3.42	−0.98
33	103.1	10.5	92.6	74	124	−50	26.8	17.2	9.6	2.6	3.17	−0.57
35	54.7	6.4	48.3	72	99	−27	22.9	18.2	4.7	2.92	3.97	−1.05
38	103.9	17.6	86.3	79	100	−21	27.2	21.6	5.6	2.75	4.37	−1.62
49	76.9	7.3	69.6	84	133	−49	29	15.4	13.6	3.19	4.93	−1.74
54	53.1	4	49.1	64	117	−53	22.2	21	1.2	2.41	6.05	−3.64
	*p* < 0.001			*p* < 0.001			*p* < 0.001			*p* < 0.001		

Abbreviations: Post‐op, post‐splenectomy; Pre‐op, pre‐splenectomy; RBC, red blood cells; RDW, red blood cell distribution width.

## 4 Discussion

Hereditary spherocytosis (HS) is the most common inherited hemolytic disease (Zamora and Schaefer 2023). Research suggests that a significant number of HS patients remain undiagnosed within populations (Ciepiela 2018), posing challenges in accurate diagnosis. Increasing awareness among clinicians about this disease would improve the prognosis of HS patients. In this study, the median age of onset in HS children was found to be 60 months (5 years), slightly earlier than the findings reported by Konca and Soker (2015). Additionally, the proportion of moderate to severe anemia in our center's patients was higher compared to previous studies (Kang et al. 2023). This may be attributed to our hospital being a regional medical center, where most children with severe clinical symptoms are presented early and receive prompt diagnosis and inclusion in this study. Previous studies have shown that approximately 75% of HS patients have autosomal dominant inheritance, while about 25% have autosomal recessive inheritance or spontaneous mutations (Iolascon et al. 2019). However, 22 patients had a family history of the disease, while the remaining 37 patients had spontaneous mutations in our study. Moreover, we detected 66 gene mutation sites, of which 58 were novel mutations, which suggest that HS patients in Chongqing, China, have unique genetic characteristics. Further expansion of the sample size in HS research is necessary to gain a better understanding.

From the perspective of the number of different mutated genes, mutations in the *ANK1* gene and *SPTB* gene are the primary pathogenic genes in HS patients at our center, which is consistent with previous research findings in other regions of China (Wang et al. 2018; Qin et al. 2020; Wu et al. 2021). In European and American populations, the mutational spectrum of hereditary spherocytosis (HS) predominantly involves the following genes: *ANK1* gene mutations, accounting for approximately 50% of HS cases, and *SPTB* gene mutations, which account for about 20% (Perrotta et al. 2008). However, in Japan or Brazil, only 5%–10% of HS cases are caused by *ANK1* mutations, which may indicate geographic differences in the distribution of *ANK1* gene mutations (Wang et al. 2021). The mutation spectrum of HS in African populations exhibits certain uniqueness. Research has found that the primary mutation sites for HS in African patients remain concentrated in the *ANK1* and *SPTB* genes. Specifically, in some regions of Africa, the mutation frequency of the *EPB42* gene in HS patients may be higher, and some mutation types exhibit higher genetic heterogeneity. Additionally, certain HS patients in African populations may exhibit different clinical manifestations, particularly in mild and recessive types of HS cases (Bianchi et al. 2012; Rojhani and Al‐Ghussain 2013). Additionally, we identified two cases of CNV mutations in the *SPTB* gene, which is a rare occurrence. This rarity may be attributed to the fact that CNV mutations can lead to fetal lethality (Griswold et al. 2011), further confirming the infrequency of CNV mutations.

In our patients, we identified only one mutation in the *SLC4A1* gene and two mutations in the *SPTA1* gene, indicating that these mutations are relatively rare in the Chinese population. This rarity may be due to the fact that heterozygous mutations in the *SLC4A1* gene, which encodes the band 3 protein, often lead to the production of partially functional proteins that mitigate the impact of the mutation, resulting in milder symptoms that are usually detected in adulthood, if at all (Tole et al. 2020). Mariani reported that band 3 deficiencies were the most common protein abnormalities (54%) in European HS patients (Mariani et al. 2008). On the other hand, heterozygous mutations in the *SPTA1* gene allow for the production of both α‐hemagglutinin and β‐hemagglutinin, which help maintain the stability of the red cell skeleton (Nussenzveig et al. 2014). However, the presence of two nonfunctional α‐spectrin alleles in the *SPTA1* gene can lead to severe challenges in fetal survival for children with homozygous mutations (Narla and Mohandas 2017; Gallagher 2005).

After comparing the clinical characteristics between the single mutation group and the multiple mutations group, we found a significant difference in gender distribution. The multiple mutations group had a higher proportion of females, and the total bilirubin levels were lower in the compound mutation group. In our research, this may suggest that gender factors may play a role in the expression of such mutations or the pathogenesis of the disease. Further research is needed to determine whether gender differences may affect the severity of the disease, clinical manifestations, or treatment response. Patients with multiple gene mutations were found to have lower total bilirubin levels, which may indicate a milder degree of hemolysis associated with this type of mutation, or the physiological mechanism of this type of mutation may differ from other types of HS. This may have guiding significance for the classification and treatment selection of HS in clinical practice. This is the first observation of such results. Further collection of HS cases is needed to validate this conclusion.

Additionally, genotype–phenotype correlation analysis of HS patients with single gene mutations showed no significant differences in laboratory indicators among patients carrying *ANK1* or *SPTB* mutation genes, which is consistent with the findings of Aggarwal et al. (2019) and Tole et al. (2020). However, stratified analysis based on the severity of anemia revealed that children carrying *ANK1* mutation genes had more severe anemia, which is consistent with previous research findings (Wu et al. 2021). Furthermore, in our study, patients carrying *ANK1* mutation genes had a higher rate of splenectomy compared to those carrying *SPTB* mutation genes. We speculate that this may be because the spleen of patients with *ANK1* mutations can more effectively clear abnormal red blood cells, leading to more severe anemia and subsequently an increased number of patients meeting the criteria for splenectomy (Wu et al. 2021).

After analyzing the mutations in different domains of the *ANK1* and *SPTB* genes and their clinical phenotypic differences, we did not find any statistically significant differences. However, a study in the Netherlands (van Vuren et al. 2019) showed that patients with mutations in the central spectrin‐binding domain of *ANK1* had more severe anemia compared to patients with mutations in other domains. This may be attributed to the unique racial and regional characteristics of Chinese children. Of course, further studies with larger sample sizes are needed to validate and explore these findings in depth.

In addition, a comparison of different types of gene mutations with clinical characteristics in HS patients revealed that patients with missense mutations had lower MCHC levels compared to patients with other mutations. In hereditary spherocytosis (HS), truncating variants (e.g., nonsense/frameshift) typically cause haploinsufficiency with pronounced membrane loss, microvesiculation, and KCl‐driven dehydration, yielding spherocytes with higher MCHC and earlier splenic clearance. By contrast, missense variants often partially preserve protein function (e.g., residual ankyrin anchoring), attenuating membrane loss and limiting dehydration; red cells may circulate longer with relatively greater hydration, resulting in a lower MCHC compared with truncating genotypes (Perrotta et al. 2008; Iolascon et al. 2019). In addition, missense changes clustered in regulatory domains (e.g., ankyrin C‐terminus or band 3 interfaces) can perturb the membrane skeleton–cytosol coupling and ion‐handling complexes, blunting the net dehydrating fluxes that elevate MCHC (Perrotta et al. 2008). Missense variants may also produce misfolded proteins retained at the membrane, promoting oxidative stress and hemoglobin denaturation/precipitation; a reduction in functional, soluble hemoglobin per cell would further lower MCHC (Perrotta et al. 2008). This may suggest that this type of mutation affects different physiological processes of red blood cells or indicates that the red blood cell membrane structure of such mutations is different from what is usually seen in HS patients. This finding may help further understand the specific role of missense mutations in the disease and may also be helpful in adjusting diagnosis and treatment strategies. However, in our study, most HS children had MCHC levels within the normal range, suggesting that routine laboratory testing of MCHC lacks specificity and sensitivity and is not an ideal indicator for diagnosing HS (Aggarwal et al. 2020; Kang et al. 2023).

## 5 Conclusion

This study analyzed the clinical and genetic mutation characteristics of 59 Chinese children with HS. We found significant heterogeneity in the clinical phenotypes and genetic mutation spectrum of HS patients. Establishing an auxiliary diagnostic system and clinical knowledge database based on the relationship between genotype and phenotype can provide important references for the diagnosis and treatment of HS patients. Future research should further expand the sample size and conduct pathogenicity validation of unidentified mutation sites.

## Author Contributions

Yang Wang and Yuzhuopu Li wrote the manuscript; Tao Liu contributed to discussion and revised the manuscript; Lan Huang and Li Xiao provided valuable research ideas; Yongjie Zhang and Yan Xiang collected the data; Jie Yu supervised the project execution.

## Funding

This work was supported by Public Welfare Project for Rare Blood Disorders in the field of Hematology in China (xyxthjb‐2023‐006) and the Open Project of the Key Laboratory of Panvascular Diseases in Hebei Province, 2024 (grant no. FXGJBKFKT2402).

## Ethics Statement

This study was conducted in accordance with the World Medical Association Declaration of Helsinki and the International Ethical Guidelines for Human Biomedical Research. Genetic screening on the entire family was performed after obtaining their signed informed consent form. The research was approved by the Medical Ethics Committee of the Children's Hospital of Chongqing Medical University (approval file number: 2021.457).

## Conflicts of Interest

The authors declare no conflicts of interest.

## Supporting information


**Data S1:** mgg370188‐sup‐0001‐supinfo.docx.

## Data Availability

The data that support the findings of this study are available from the corresponding author upon reasonable request.

## References

[mgg370188-bib-0029] Aggarwal, A. , M. Jamwal , P. Sharma , et al. 2019. “Deciphering molecular heterogeneity of Indian families with hereditary spherocytosis using targeted next‐generation sequencing: first South Asian study.” Br. J. Haematol 188: 784–795. 10.1111/bjh.16244. 31602632

[mgg370188-bib-0001] Aggarwal, A. , M. Jamwal , P. Sharma , et al. 2020. “Deciphering Molecular Heterogeneity of Indian Families With Hereditary Spherocytosis Using Targeted Next‐Generation Sequencing: First South Asian Study.” British Journal of Haematology 188, no. 5: 784–795. 10.1111/bjh.16244. 31602632

[mgg370188-bib-0002] Bianchi, P. , E. Fermo , C. Vercellati , and A. P. Marcello . 2012. “Molecular Characterization of 35 Italian Patients Affected by Hereditary Spherocytosis: Association of Known and New Ankyrin Mutations With Clinical Severity.” Haematologica 97, no. 4: 516–523. 22058213 10.3324/haematol.2011.052845 PMC3347664

[mgg370188-bib-0003] Bolton‐Maggs, P. H. , J. C. Langer , A. Iolascon , P. Tittensor , and M. J. King . 2012. “Guidelines for the Diagnosis and Management of Hereditary Spherocytosis–2011 Update.” British Journal of Haematology 156, no. 1: 37–49. 10.1111/j.1365-2141.2011.08921.x. 22055020

[mgg370188-bib-0004] Butorac, A. I. , D. K. Baraba , G. Palcrvski , and J. Roganovic . 2018. “An Infant With Unusually High Unconjugated Hyperbilirubinemia due to Coexistence of Hereditary Spherocytosis and Gilbert Syndrome.” Journal of Pediatric Hematology/Oncology 40, no. 2: 127–128. 10.1097/mph.0000000000001025. 29200157

[mgg370188-bib-0005] Ciepiela, O. 2018. “Old and New Insights Into the Diagnosis of Hereditary Spherocytosis.” Annals of Translational Medicine 6, no. 17: 339. 10.21037/atm.2018.07.35. 30306078 PMC6174190

[mgg370188-bib-0028] Gallagher, P. G. 2005. “Hematologically important mutations: Ankyrin variants in hereditary spherocytosis.” Blood Cells, Molecules, & Diseases 35: 34–37. 10.1016/j.bcmd.2005.08.008 16223590

[mgg370188-bib-0006] Griswold, A. J. , D. Ma , S. J. Sacharow , et al. 2011. “A *de Novo* 1.5 Mb Microdeletion on Chromosome 14q23.2–23.3 in a Patient With Autism and Spherocytosis.” Autism Research 4: 221–227. 10.1002/aur.186. 21360829 PMC3110642

[mgg370188-bib-0007] Iolascon, A. , I. Andolfo , and R. Russo . 2019. “Advances in Understanding the Pathogenesis of Red Cell Membrane Disorders.” British Journal of Haematology 187, no. 1: 13–24. 10.1111/bjh.16126. 31364155

[mgg370188-bib-0008] Kang, M. , H. Li , J. Zhu , L. Zhu , Y. Hong , and Y. Fang . 2023. “Clinical Manifestations of 17 Chinese Children With Hereditary Spherocytosis Caused by Novel Mutations of the *ANK1* Gene and Phenotypic Analysis.” Frontiers in Genetics 14: 1088–1985. 10.3389/fgene.2023.1088985. PMC9929461 36816036

[mgg370188-bib-0009] Konca, Ç. , and M. Soker . 2015. “Hereditary Spherocytosis in Childhood: Clinical and Laboratory Study.” Turkish Journal of Hematology 32, no. 2: 147–152.

[mgg370188-bib-0010] Li, S. , P. Guo , L. Mi , et al. 2022. “A Novel *SPTB* Mutation Causes Hereditary Spherocytosis via Loss‐Of‐Function of β‐Spectrin.” Annals of Hematology 101: 731–738. 10.1007/s00277-022-04773-3. 35099593

[mgg370188-bib-0011] Mariani, M. , W. Barcellini , C. Vercellati , et al. 2008. “Clinical and Hematologic Features of 300 Patients Affected by Hereditary Spherocytosis Grouped According to the Type of the Membrane Protein Defect.” Haematologica 93, no. 9: 1310–1317. 10.3324/haematol.12546. 18641031

[mgg370188-bib-0012] Narla, J. , and N. Mohandas . 2017. “Red Cell Membrane Disorders.” International Journal of Laboratory Hematology 39, no. Suppl 1: 47–52. 10.1111/ijlh.12657. 28447420

[mgg370188-bib-0013] Nussenzveig, R. H. , R. D. Christensen , J. T. Prchal , H. M. Yaish , and A. M. Agarwal . 2014. “Novel α‐Spectrin Mutation in Trans With α‐SpectrinLEPRA Causing Severe Neonatal Jaundice From Hereditary Spherocytosis.” Neonatology 106, no. 4: 355–357. 10.1159/000365586. 25277063

[mgg370188-bib-0014] Park, J. , D. C. Jeong , J. Yoo , et al. 2016. “Mutational Characteristics of *ANK1* and *SPTB* Genes in Hereditary Spherocytosis.” Clinical Genetics 90: 69–78. 10.1111/cge.12749. 26830532

[mgg370188-bib-0015] Perrotta, S. , P. G. Gallagher , and N. Mohandas . 2008. “Hereditary Spherocytosis.” Lancet 372: 1411–1426. 10.1016/S0140-6736(08)61588-3. 18940465

[mgg370188-bib-0016] Qin, L. , Y. Nie , H. Zhang , et al. 2020. “Identification of New Mutations in Patients With Hereditary Spherocytosis by Next Generation Sequencing.” Journal of Human Genetics 65: 427–434. 10.1038/s10038-020-0724-z. 31980736

[mgg370188-bib-0017] Richards, S. , N. Aziz , S. Bale , et al. 2015. “Standards and Guidelines for the Interpretation of Sequence Variants: A Joint Consensus Recommendation of the American College of Medical Genetics and Genomics and the Association for Molecular Pathology.” Genetics in Medicine 17, no. 5: 405–424. 10.1038/gim.2015.300. 25741868 PMC4544753

[mgg370188-bib-0018] Rojhani, A. , and L. Al‐Ghussain . 2013. “Hereditary Spherocytosis and Genetic Mutations in African Populations.” African Journal of Hematology 8, no. 2: 214–223.

[mgg370188-bib-0019] Tole, S. , P. Dhir , J. Pugi , et al. 2020. “Genotype‐Phenotype Correlation in Children With Hereditary Spherocytosis.” British Journal of Haematology 191: 486–496. 10.1111/bjh.16750. 32436265

[mgg370188-bib-0020] van Vuren, A. , B. van der Zwaag , R. Huisjes , et al. 2019. “The Complexity of Genotype‐Phenotype Correlations in Hereditary Spherocytosis: A Cohort of 95 Patients.” HemaSphere 3, no. 4: e276. 10.1097/HS9.0000000000000276. 31723846 PMC6745925

[mgg370188-bib-0021] Wang, C. , Y. Cui , Y. Li , X. Liu , and J. Han . 2015. “A Systematic Review of Hereditary Spherocytosis Reported in Chinese Biomedical Journals From 1978 to 2013 and Estimation of the Prevalence of the Disease Using a Disease Model.” Intractable & Rare Diseases Research 4: 76–81. 10.5582/irdr.2015.01002. 25984425 PMC4428190

[mgg370188-bib-0022] Wang, D. , L. Song , L. Shen , et al. 2021. “Mutational Characteristics of Causative Genes in Chinese Hereditary Spherocytosis Patients: A Report on Fourteen Cases and a Review of the Literature.” Frontiers in Pharmacology 12: 644352. 10.3389/fphar.2021.644352. 34335240 PMC8322660

[mgg370188-bib-0023] Wang, R. , S. Yang , M. Xu , et al. 2018. “Exome Sequencing Confirms Molecular Diagnoses in 38 Chinese Families With Hereditary Spherocytosis.” Science China. Life Sciences 61: 947–953. 10.1007/s11427-017-9232-6. 29572776

[mgg370188-bib-0024] Wu, C. , T. Xiong , Z. Xu , et al. 2021. “Preliminary Study on the Clinical and Genetic Characteristics of Hereditary Spherocytosis in 15 Chinese Children.” Frontiers in Genetics 12: 652376. 10.3389/fgene.2021.652376. 33868383 PMC8044778

[mgg370188-bib-0025] Yamamoto, K. S. , T. Utshigisawa , H. Ogura , et al. 2022. “Clinical and Genetic Diagnosis of Thirteen Japanese Patients With Hereditary Spherocytosis.” Human Genome Variation 9, no. 1: 1. 10.1038/s41439-021-00179-1. 35022413 PMC8755803

[mgg370188-bib-0026] Yang, N. , S. Wu , and J. Yan . 2019. “Structural Variation in Complex Genome: Detection, Integration and Function.” Science China. Life Sciences 62, no. 8: 1098–1100. 10.1007/s11427-019-9664-4. 31376014

[mgg370188-bib-0027] Zamora, E. A. , and C. A. Schaefer . 2023. “Hereditary Spherocytosis.” In StatPearls. StatPearls Publishing. 30969619

