# Supplementary material for: Clinical Characteristics and Gene Mutations of Hereditary Spherocytosis in 59 Chinese Children
Source: Mol Genet Genomic Med. 2026 Feb 21;14(3):e70188. doi: 10.1002/mgg3.70188 (PMC12927912; doi:10.1002/mgg3.70188)
Supplement: Supplementary file 1 — Data S1: mgg370188‐sup‐0001‐supinfo.docx. [file MGG3-14-e70188-s001.docx]

Supplementary Material

1 Supplementary Data

| Supplementary Data 1 Baseline clinical and hematologic characteristics of the study cohort (n = 59). Listed are age at diagnosis (months), sex, history of blood transfusion, presence of splenomegaly, history of splenectomy and age at splenectomy (months), and laboratory indices at presentation | | | | | | | | | | | | | | |
| --- | --- | --- | --- | --- | --- | --- | --- | --- | --- | --- | --- | --- | --- | --- |
| **ID** | | **Month-old at diagnosis** | **Gender** | **Blood transfusion** | **Splenomegaly** | **Splenectomy** | **Month-old of splenectomy** | **Total bilirubin(umol/L)** | **RDW (%)** | **RBC(*10^12/L)** | **Ret** | **MCV (fL)** | **MCHC(g/L)** | **Hb(g/L)** |
| 1 | 11 | | female | Y | Y | N | -------- | 16.4 | 25 | 2.55 | 0.084 | 86.7 | 317 | 70 |
| 2 | 60 | | male | Y | Y | N | -------- | 43.4 | 21 | 3.31 | 0.067 | 84 | 335 | 93 |
| 3 | 0.33 | | male | Y | Y | N | -------- | 83.4 | 20.8 | 3.76 | 0.065 | 77.9 | 352 | 69 |
| 4 | 24 | | male | Y | Y | N | -------- | 25.3 | 16.9 | 3.67 | 0.048 | 83.9 | 334 | 103 |
| 5 | 0.53 | | male | Y | Y | N | -------- | 171.5 | 16.8 | 2.45 | 0.031 | 82.4 | 356 | 72 |
| 6 | 60 | | male | Y | Y | N | -------- | 63.4 | 21 | 3.08 | 0.077 | 73.3 | 355 | 90 |
| 7 | 5 | | male | N | Y | N | -------- | 73 | 25.3 | 2.97 | 0.151 | 84.5 | 315 | 79 |
| 8 | 72 | | female | N | Y | N | -------- | 49.1 | 25 | 3.54 | 0.022 | 81.4 | 347 | 100 |
| 9 | 60 | | female | Y | Y | N | -------- | 43.3 | 22.4 | 2.98 | 0.082 | 88.3 | 327 | 84 |
| 10 | 22 | | female | N | Y | N | -------- | 30.1 | 26.5 | 3.05 | 0.015 | 88.9 | 328 | 89 |
| 11 | 141 | | female | Y | Y | N | -------- | 82.1 | 24.1 | 3.17 | 0.013 | 83.9 | 335 | 89 |
| 12 | 131 | | male | Y | Y | Y | 134 | 149 | 27.6 | 3.56 | 0.018 | 77.5 | 322 | 89 |
| 13 | 30 | | female | N | Y | N | -------- | 70 | 26.7 | 2.87 | 0.109 | 82.9 | 328 | 78 |
| 14 | 63 | | male | Y | Y | Y | 80 | 18.2 | 23.2 | 3.45 | 0.09 | 78.6 | 325 | 88 |
| 15 | 135 | | female | Y | Y | N | -------- | 16.4 | 17.3 | 1.26 | 0.52 | 69.8 | 331 | 30 |
| 16 | 6 | | male | N | N | N | -------- | 56 | 27.3 | 2.89 | 0.012 | 85.5 | 324 | 80 |
| 17 | 46 | | male | Y | Y | N | -------- | 24.2 | 30.4 | 2.2 | 0.082 | 77.7 | 304 | 52 |
| 18 | 25 | | female | Y | Y | N | -------- | 33.4 | 26.1 | 3.05 | 0.077 | 80 | 316 | 77 |
| 19 | 100 | | female | Y | Y | Y | 100 | 72.8 | 41 | 2.43 | 0.04 | 82.7 | 234 | 47 |
| 20 | 129 | | female | Y | Y | N | -------- | 74.2 | 20 | 2.49 | 0.109 | 86.3 | 344 | 74 |
| 21 | 11.8 | | male | Y | Y | Y | 102 | 61.1 | 23.4 | 3.8 | 0.06 | 77.9 | 331 | 98 |
| 22 | 1.9 | | male | Y | Y | N | -------- | 66.4 | 15.6 | 2.57 | 0.064 | 85.6 | 341 | 75 |
| 23 | 0 | | female | Y | N | N | -------- | 338.9 | 15 | 2.43 | 0.091 | 81.1 | 305 | 60 |
| 24 | 67 | | male | Y | Y | N | -------- | 35.8 | 22.9 | 2.29 | 0.107 | 90.4 | 329 | 68 |
| 25 | 101 | | female | Y | Y | Y | 102 | 25.4 | 19.8 | 3.32 | 0.05 | 78.9 | 336 | 88 |
| 26 | 180 | | female | Y | Y | Y | 180 | 157.6 | 25.8 | 3.19 | 0.191 | 82.1 | 340 | 89 |
| 27 | 85 | | female | Y | Y | Y | -------- | 80.8 | 27.8 | 2.44 | 0.14 | 80.3 | 311 | 61 |
| 28 | 86 | | female | N | Y | N | -------- | 48.7 | 27.1 | 2.71 | 0.119 | 82.3 | 314 | 70 |
| 29 | 71 | | male | N | N | N | -------- | 4 | 15.7 | 4.13 | 0.014 | 78.5 | 324 | 105 |
| 30 | 32 | | female | Y | Y | N | -------- | 83 | 27.9 | 1.98 | 0.087 | 72.7 | 333 | 48 |
| 31 | 152 | | female | Y | Y | N | -------- | 90.7 | 18.2 | 2.79 | 0.121 | 87.1 | 362 | 88 |
| 32 | 87 | | male | Y | Y | N | -------- | 61.1 | 24.7 | 2.77 | 0.113 | 85.2 | 335 | 79 |
| 33 | 146 | | female | Y | Y | Y | 161 | 103.1 | 26.8 | 2.6 | 0.087 | 88.8 | 320 | 74 |
| 34 | 0.83 | | female | Y | Y | N | -------- | 66.3 | 17 | 2.06 | 0.037 | 81.6 | 363 | 61 |
| 35 | 90 | | female | Y | Y | Y | 126 | 54.7 | 22.9 | 2.92 | 0.067 | 72.9 | 338 | 72 |
| 36 | 27 | | female | Y | Y | N | -------- | 38.6 | 30.7 | 2.3 | 0.216 | 90 | 314 | 65 |
| 37 | 9.5 | | female | Y | N | N | -------- | 28.9 | 21.7 | 3.13 | 0.145 | 83.4 | 330 | 86 |
| 38 | 92 | | female | Y | Y | Y | 115 | 103.9 | 27.2 | 2.75 | 0.148 | 85.1 | 338 | 79 |
| 39 | 24 | | male | Y | N | N | -------- | 85 | 18.7 | 2.28 | 0.067 | 79.8 | 335 | 61 |
| 40 | 138 | | female | Y | Y | N | -------- | 98.2 | 20 | 2.52 | 0.124 | 83.7 | 318 | 67 |
| 41 | 47 | | female | N | N | N | -------- | 23.3 | 21.1 | 3.05 | 0.062 | 84.9 | 320 | 83 |
| 42 | 49 | | male | N | Y | N | -------- | 44.4 | 19.7 | 3.11 | 0.132 | 86.5 | 309 | 83 |
| 43 | 9 | | male | Y | N | N | -------- | 35.8 | 22.6 | 3.35 | 0.051 | 81.8 | 339 | 93 |
| 44 | 97 | | male | Y | N | N | -------- | 8.7 | 26.3 | 3.36 | 0.038 | 75.9 | 302 | 77 |
| 45 | 2.9 | | female | Y | N | N | -------- | 27.2 | 18.1 | 2.39 | 0.121 | 81.6 | 333 | 65 |
| 46 | 3.2 | | female | Y | N | N | -------- | 22.8 | 19.4 | 3.25 | 0.0558 | 86.2 | 336 | 94 |
| 47 | 14 | | male | Y | N | N | -------- | 48 | 28.5 | 1.96 | 0.194 | 86.2 | 302 | 51 |
| 48 | 53 | | female | Y | Y | N | -------- | 73.5 | 24.8 | 2.61 | 0.077 | 84.7 | 335 | 74 |
| 49 | 55 | | female | Y | Y | Y | 62 | 76.9 | 29 | 3.19 | 0.131 | 90 | 293 | 84 |
| 50 | 65 | | male | Y | Y | N | -------- | 88.3 | 27.7 | 3.14 | 0.082 | 74.5 | 312 | 73 |
| 51 | 112 | | male | N | Y | N | -------- | 94 | 23.3 | 2.84 | 0.261 | 91.5 | 335 | 87 |
| 52 | 109 | | male | Y | Y | N | -------- | 94 | 24 | 2.93 | 0.019 | 79.2 | 315 | 73 |
| 53 | 46 | | female | Y | Y | N | -------- | 79 | 25.5 | 3.22 | 0.0852 | 83.2 | 321 | 86 |
| 54 | 104 | | male | Y | Y | Y | 104 | 53.1 | 22.2 | 2.41 | 0.1147 | 94.6 | 281 | 64 |
| 55 | 39 | | female | N | Y | N | -------- | 65.8 | 27.7 | 2.96 | 0.1221 | 86.8 | 323 | 83 |
| 56 | 98 | | male | Y | Y | N | -------- | 62.7 | 24.4 | 2.81 | 0.128 | 88.3 | 335 | 75 |
| 57 | 84 | | male | Y | Y | N | -------- | 49 | 19.6 | 3.68 | 0.08 | 79.6 | 348 | 102 |
| 58 | 1.9 | | female | Y | Y | N | -------- | 164.2 | 20.3 | 2.76 | 0.127 | 93.6 | 308 | 77 |
| 59 | 102 | | male | Y | Y | N | -------- | 59.9 | 22.1 | 3.3 | 0.063 | 78.8 | 342 | 89 |

#

2 Supplementary Tables

| **Supplementary Table 1** Summary of rare variants identified in the study cohort. For each proband (ID), the gene, genomic location, cDNA and predicted protein change (HGVS), zygosity (Status), ACMG classification, inheritance, variant type, and whether the variant is previously reported or novel are shown. Multiple rows for the same ID indicate multiple variants in the same individual; literature references are provided in brackets (PMID) in the Known column. | | | | | | | | | |
| --- | --- | --- | --- | --- | --- | --- | --- | --- | --- |
| **ID** | **Gene** | **Location** | **cDNA change** | **Protein effect** | **Status** | **ACMG Classification** | **Inheritance** | **Type** | **Known/ novel** |
| 1 | *SPTB* | chr14:65246625 | c.4291C>T | p.R1431* | het | Pathogenic | De novo | Nonsense | Reported [PMID26830532] |
| 2 | *SPTB* | chr14:65239585 | c.5266C>T | p.R1756* | het | Pathogenic | De novo | Nonsense | Reported [PMID26830532] [PMID19538529] |
| 3 | *SPTB* | chr14:65289758 | c.48_55dup | p.I19Tfs*30 | het | Pathogenic | De novo | Frameshift | Novel |
| 4 | *ANK1* | chr8:41582047 | c.737C>A | p.A246E | het | Likely pathogenic | Paternal | Missense | Novel |
| 5 | *SPTB* | chr14:65241950 | c.4735C>T | p.R1579* | het | Pathogenic | De novo | Nonsense | Reported [PMID29572776] |
| 6 | *SPTB* | chr14:65241215 | c.4873C>T | p.R1625* | het | Likely pathogenic | De novo | Nonsense | Reported [PMID27292444] |
| 7 | *ANK1* | chr8:41573253 | c.1618delC | p.L540Cfs*26 | het | Likely pathogenic | De novo | Frameshift | Novel |
| 8 | *SPTB* | chr14:65252320 | c.3790C>T | p.Q1264* | het | Likely pathogenic | De novo | Nonsense | Novel |
| 9 | *SPTB* | chr14:65239585 | c.5266C>T | p.R1756* | het | Likely pathogenic | Maternal | Nonsense | Reported [PMID19538529] |
|  | *SPTA1* | chr1:158648201 | c.802C>T | p.R268* | het | Likely pathogenic | Paternal | Nonsense | Novel |
| 10 | *ANK1* | chr8:41547801 | c.4171G>T | p.E1391* | het | Likely pathogenic | De novo | Nonsense | Novel |
|  | *ANK1* | chr8:41550356 | c.3791T>C | p.V1264A | het | Likely pathogenic | Maternal | Missense | Novel |
| 11 | *ANK1* | chr8:41561572 | c.2381delA | p.N794Tfs*13 | het | Likely pathogenic | De novo | Frameshift | Novel |
| 12 | *ANK1* | chr8:41513274 | c.5668-2_5668-1insAAGA | - | het | Uncertain | De novo | Splicing | Novel |
| 13 | *ANK1* | chr8:41521276 | c.5518-16C>G | - | het | Uncertain | Paternal | Splicing | Novel |
|  | *ANK1* | chr8:41546092 | c.4243G>A | p.D1415N | het | Likely pathogenic | Maternal | Missense | Novel |
| 14 | *ANK1* | chr8:41543670-41546107 | 35-37 exonic deletion | - | het | x1 | De novo | CNV | Novel |
| 15 | *ANK1* | chr8:41552280 | c.3280C>T | p.R1094X | het | Pathogenic | De novo | Nonsense | Reported [PMID:12899723] |
| 16 | *SPTB* | chr14:65235837 | c.5938-1G>C | - | het | Pathogenic | De novo | Splicing | Novel |
| 17 | *ANK1* | chr8:41552759 | c.3051G>A | p.W1017X | het | Likely pathogenic | Maternal | Missense | Novel |
| 18 | *ANK1* | chr8:41547762-41547763 | c.4086delC | p.M1363fs*43 | het | Likely pathogenic | Maternal | Frameshift | Novel |
| 19 | *ANK1* | chr8:41583332 | c.559C>T | p.R187C | het | Likely pathogenic | Maternal | Missense | Novel |
| 20 | *SPTB* | chr14:65234417 | c.6183G>A | p.W2061X | het | Likely pathogenic | Maternal | Nonsense | Novel |
| 21 | *ANK1* | chr8:41583333-41583333 | c.558delG | p.R187Afs*66 | het | pathogenic | De novo | Frameshift | Novel |
| 22 | *SPTB* | chr8:65239646 | c.5205delT | p.F1735fs | het | Pathogenic | De novo | Frameshift | Novel |
| 23 | *ANK1* | chr8:41571765 | c.1702_1709del | p.568_570del | het | Pathogenic | De novo | Frameshift | Novel |
|  | *ANK1* | chr8:41571765 | c.1801_1808del | p.601_603del | het | Pathogenic | De novo | Frameshift | Novel |
| 24 | *SPTB* | chr14:65271716 | c.241G>T | p.D81Y | het | Likely pathogenic | Maternal | Missense | Novel |
| 25 | *ANK1* | chr8:41583451 | c.440C>G | p.P147R | het | Likely pathogenic | De novo | Missense | Novel |
|  | *ANK1* | chr8:41583451 | c.539C>G | p.P180R | het | Likely pathogenic | De novo | Missense | Novel |
| 26 | *SPTB* | chr14:65271802 | c.155G>A | p.R52Q | het | Likely pathogenic | De novo | Missense | Reported [PMID: 24193021] |
| 27 | *ANK1* | chr8:41591533 | c.184delG | p.V62fs | het | Likely pathogenic | Paternal | Frameshift | Novel |
| 28 | *ANK1* | chr8:41552236 | c.3200dupC | p.S1067fs | het | Pathogenic | De novo | Frameshift | Novel |
| 29 | *SPTA1* | chr1:15862452 | c.2909C>A | p.A970D | het | Likely pathogenic | Paternal | Missense | Reported [PMID: 8370581] |
| 30 | *ANK1* | chr8:41552760 | c.3050G>A | p.W1017X | het | Pathogenic | De novo | Nonsense | Novel |
| 31 | *SPTB* | chr14:65252664 | c.3567delC | p.Y1189X | het | Pathogenic | Paternal | Frameshift | Novel |
| 32 | *SPTB* | chr14:65260469 | c.1912C>T | p.R638X | het | Pathogenic | De novo | Nonsense | Novel |
| 33 | *ANK1* | chr8:41561975 | c.2113delC | p.L70 5fs | het | Pathogenic | Paternal | Frameshift | Novel |
| 34 | *SPTB* | chr14:65246649 | c.4267C>T | p.R1423X | het | Pathogenic | De novo | Nonsense | Novel |
| 35 | *SPTB* | chr14:65246513 | c.4403delG | p.G1468fs | het | Pathogenic | De novo | Frameshift | Novel |
| 36 | *SPTB* | chr14:65252671 | c.3562-2A>G | - | het | Pathogenic | Maternal | Splicing | Novel |
|  | *SPTB* | chr14:65220501 | c.6354_6356del | p.2118_2119del | het | Pathogenic | Paternal | Frameshift | Novel |
|  | *SPTB* | chr14:65220373 | c.6484G>A | p.E2162K | het | Pathogenic | Maternal | Missense | Novel |
| 37 | *ANK1* | chr8:41552280 | c.3157C>T | p.R1053X | het | Pathogenic | De novo | Nonsense | Novel |
| 38 | *SPTB* | chr14:65236342 | c.5902_5903insGCCTGCCTGGAGCTTGGCGAG | p.S1968fs | het | Pathogenic | De novo | Frameshift | Novel |
| 39 | *SPTB* | chr14:65253292 | c.3391C>T | p.Q1131X | het | Pathogenic | De novo | Nonsense | Novel |
| 40 | *ANK1* | chr8:41551574 | c.3374delA | p.Q1125fs | het | Pathogenic | De novo | Frameshift | Novel |
| 41 | *SPTB* | chr14:65270314-65271818 | - | - | x1 | Likely pathogenic | Maternal | CNV | Novel |
| 42 | *ANK1* | chr8:41551430 | c.3518T>C | p.L1173P | het | Pathogenic | De novo | Missense | Novel |
| 43 | *SPTB* | chr14:65253416 | c.3267delG | p.M1089fs | het | Pathogenic | De novo | Frameshift | Novel |
| 44 | *SPTA1* | chr1:158583632 | c.6868C>T | p.R2290C | het | Likely pathogenic | Maternal | Missense | Novel |
| 45 | *ANK1* | chr8:41581135 | c.728T>G | p.L243R | het | Pathogenic | De novo | Missense | Novel |
| 46 | *SPTB* | chr14:65259970 | c.2410dupC | p.Q804fs | het | Pathogenic | De novo | Frameshift | Novel |
| 47 | *ANK1* | chr8:41554292 | c.2761-1G>A | - | het | Pathogenic | De novo | Splicing | Novel |
| 48 | *SPTB* | chr14:65268947 | c.563C>T | p.A188V | het | Pathogenic | De novo | Missense | Novel |
| 49 | *ANK1* | chr8:41580711 | c.841C>T | p.R281X | het | Pathogenic | De novo | Missense | Novel |
| 50 | *SPTB* | chr14:65253379 | c.3304C>T | p.Q1102X | het | Pathogenic | Paternal | Nonsense | Novel |
| 51 | *ANK1* | chr8:41550257 | c.3767T>C | p.L1256P | het | Likely pathogenic | Paternal | Missense | Novel |
| 52 | *ANK1* | chr8:41581090 | c.773T>C | p.L258P | het | Likely pathogenic | Paternal | Missense | Novel |
| 53 | *SPTB* | chr14:65270325-65271808 | 2-3 exonic deletion | - | x1 | Likely pathogenic | Maternal | CNV | Novel |
| 54 | *SLC4A1* | chr17:42335071 | c.1387G>A | p.G463s | het | Likely pathogenic | Paternal | Missense | Novel |
| 55 | *SPTB* | chr14:65239480 | c.5371C>T | p.Q1791X | het | Pathogenic | Paternal | Nonsense | Novel |
| 56 | *ANK1* | chr8:41585500 | c.253G>C | p.A85P | het | Likely pathogenic | Paternal | Missense | Novel |
| 57 | *SPTB* | chr14:65270500 | c.301-2A>G | - | het | Pathogenic | De novo | Splicing | Novel |
| 58 | *ANK1* | chr8:41575120 | c.1305+2T>- | - | het | Pathogenic | De novo | Splicing | Novel |
| 59 | *ANK1* | chr8:41575120 | c.1305+2T>A | - | het | Pathogenic | De novo | Splicing | Novel |
| Notes: het = heterozygous. Genomic coordinates are given as chromosome:position; reference genome build as specified in Methods. Protein changes use * to denote a stop codon and fs for frameshift; splice-site variants have “—” for protein effect. CNV = copy-number variant; “x1” denotes a heterozygous deletion spanning the indicated interval/exons. De novo, paternal, and maternal refer to segregation results. Known/novel indicates prior reports; PMIDs cite the source papers. | | | | | | | | | |

**Supplementary Tables 2** Comparison between *ANK1* mutation sites and clinical manifestations.

| *ANK1* anchoring domain | yellow region, n=11 | blue region, n=8 | *P*-Value |
| --- | --- | --- | --- |
| Hb (g/L), Med (range) | 74(47-103) | 69(30-87) | 0.809 |
| MCV (FL), Med (range) | 83.9(77.9-90.0) | 82.9(69.8-91.5) | 0.086 |
| MCHC (g/L), Med (range) | 315(234-335) | 324(304-335) | 0.299 |
| Retics (%), Med (range) | 8.7(1.3-15.1) | 12.8(8.2-52) | 0.065 |
| TBIL (μmol/L), Med (range) | 73(25.3-103.1) | 46.6(16.4-98.2) | 0.173 |
| The yellow region represents the N-terminal membrane protein binding domain, the blue region represents the central domain. | | | |

**Supplementary Tables 3** Comparison between *SPTB* mutation sites and clinical manifestations.

| *STPB* domain | blue region*, n=2* | gray region*, n=15* | dark green region*, n=3* | *P-*Value |
| --- | --- | --- | --- | --- |
| Hb (g/L), Med (range) | 71(68-74) | 79(61-100) | 83(75-93) | 0.502 |
| MCV (FL), Med (range) | 87.6(84.7-90.4) | 81.8(72.9-87.1) | 85.6(84-86.8) | 0.102 |
| MCHC (g/L), Med (range) | 332(329-335) | 339(312-363) | 335(323-341) | 0.458 |
| Retics (%), Med (range) | 9.2(7.7-10.7) | 6.7(2.2-19.1) | 7.5(6.4-12.2) | 0.94 |
| TBIL (μmol/L), Med (range) | 54.7(35.8-73.5) | 63.3(16.4-171.5) | 65.8(43.3-66.4) | 0.645 |
| The blue region represents the actin binding domain, the gray region represents parts of the spectrin repeats domain, the dark green region represents the ankyrin binding domain. | | | | |

# Supplementary Tables 4 Comparison of Mutated Genes and Mutation Patterns of Different Membrane Proteins with Clinical Symptoms in Children

| Characteristic | Mild | Moderate | Severe | *P-*Value |
| --- | --- | --- | --- | --- |
| Gene |  |  |  | 0.041 |
| *ANK1*, n | 2 | 18 | 5 |  |
| *SPTB*, n | 5 | 20 | 0 |  |
| Type |  |  |  | 0.880 |
| Nonsense, n | 2 | 10 | 2 |  |
| Missense, n | 2 | 11 | 2 |  |
| Splicing, n | 1 | 4 | 1 |  |
| Frameshift, n | 3 | 12 | 0 |  |
| CNV, n | 0 | 3 | 0 |  |
| CNV, copy number polymorphism. | | | | |

# Supplementary Tables 5 Comparison of Genetic Patterns of Different Membrane Proteins with Clinical Symptoms in Children

| Characteristic | Mild | Moderate | Severe | *P-*Value |
| --- | --- | --- | --- | --- |
| Inheritance, n |  |  |  | 0.410 |
| De novo, n | 6 | 27 | 4 |  |
| Paternal, n | 2 | 10 | 0 |  |
| Maternal, n | 0 | 8 | 2 |  |
